# Empathizing-systemizing cognitive styles: Effects of sex and academic degree

**DOI:** 10.1371/journal.pone.0194515

**Published:** 2018-03-26

**Authors:** Rachel Kidron, Leon Kaganovskiy, Simon Baron-Cohen

**Affiliations:** 1 College of Arts and Letters, Stevens Institute of Technology, Hoboken, New Jersey, United States of America; 2 Department of Psychology, Touro College and University system, New York, New York, United States of America; 3 Mathematics Department, Touro College and University system, New York, New York, United States of America; 4 Autism Research Centre, Department of Psychiatry, University of Cambridge, Cambridge, United Kingdom; 5 Cambridge Lifespan Asperger Syndrome Service (CLASS) Clinic, Cambridgeshire and Peterborough NHS Foundation Trust, Cambridge, United Kingdom; Nagoya Mental Clinic, JAPAN

## Abstract

This study tests if the drives to empathize (E) and systemize (S), measured by the Systemizing Quotient-Revised (SQ-R) and Empathy Quotient (EQ), show effects of sex and academic degree. The responses of 419 students from the Humanities and the Physical Sciences were analyzed in terms of the E-S theory predictions. Results confirm that there is an interaction between sex, degree and the drive to empathize relative to systemize. Female students in the Humanities on average had a stronger drive to empathize than to systemize in comparison to males in the Humanities. Male students in the Sciences on average had a stronger drive to systemize than to empathize in comparison to females in the Sciences. Finally, students in the sciences on average had a stronger drive to systemize more than to empathize, irrespective of their sex. The reverse is true for students in the Humanities. These results strongly replicate earlier findings.

## Introduction

People differ to the extent that they relate to social world or to rule-governed systems of the physical world. The empathizing–systemizing (E-S) theory explains these differences in terms of the two orthogonal dimensions. Empathizing is the drive to identify others’ emotions and thoughts and to respond to them with an appropriate emotion [1–3]. Systemizing is the drive to analyze systems in terms of the rules that govern them, and the drive to construct systems [1–2, 4]. Although stated as drives, systemizing and empathizing are also understood as cognitive styles and behavioral tendencies. Systemizing requires attention to detail as a prerequisite to understand systems, but also the ability to integrate them into functional wholes [5]. Empathizing, in addition to the skill of interpreting verbal and nonverbal signals and inferring the person’s underlying mental state, involves this information triggering an appropriate emotional reaction. Both drives are manifested in everyday situations, and the stronger the drive, the more it is employed.

The E-S theory claims that individual differences in the drive to empathize relative to the drive to systemize result from differences in brain structure and connectivity. There are brains that are mainly hard-wired for empathizing and others mainly hard-wired for systemizing. Specifically, 5 “brain types” are distinguished. Type E individuals are driven to empathize more than to systemize (E > S). Type S individuals are driven more to systemize than to empathize (S > E). Type B individuals, also called the “balanced brain”, are equally driven to empathize and to systemize (E = S). Extreme type E individuals empathize to an above average level whilst their drive to systematize is either average or even below average. They show high levels of inter-subjectivity but at the same time may show degrees of “system-blindness”. Extreme type S individuals systemize either at average or above average levels, are highly capable of analyzing and constructing systems, but their empathizing drive is below average, so are less capable to infer the mental states of others. They show degrees of “mind-blindness” [1, 4–7]. According to the theory, what influences brain organization into these “brain types” includes prenatal testosterone levels [5, 7]. Higher levels of prenatal testosterone were found to be correlated with a stronger drive to systemize and a lower drive to empathize [8–11]. Levels of prenatal testosterone are on average, higher in the male fetus than in the female fetus, so these hormone levels may in part explain the typical sex differences observed in empathy and systemizing [8–11].

To measure the hypothetical drives to systemizing and to empathizing and also to operationally define “brain types”, two self-report questionnaires were designed: the Systemizing Quotient-Revised (SQ-R) and the Empathy Quotient (EQ). The SQ-R measures the ability to systemize across various domains and situations [12]. Originally the SQ was composed of 40 scoring items and 20 filler items [4] and then was revised to include 35 more items. The revised version (SQ-R) includes 75 items that cover more systems; the items are sex-neutral and overall the questionnaire has better statistical properties [12]. The EQ measures the cognitive and the affective aspects of empathy in various situations [3, 13]. Two versions exist. The original includes 40 scoring items and 20 filler items [3, 13] and the second includes just the 40 scoreable items [14]. Assigning individuals into the 5 “brain types” is done by calculating the *discrepancy* between scores on the EQ and SQ-R questionnaires (E–S discrepancy), rather than by summing the scores or comparing individuals on the total score of each questionnaire alone [15].

In support of the prediction of the E-S theory regarding the neural basis of the drives, studies using data based on the questionnaires have found that the drive to systemize relative to the drive to empathize is associated with increase in gray matter and neural activity in specific brain areas [16–18]. These findings do not imply of course that the neural basis of systemizing and empathizing exist at birth or that there is a cause and effect relationship between brain organization and the drives to systemizing and to empathizing. They are simply another way of validating these two.

Based on the E-S theory it is assumed that individuals in different academic disciplines would differ in their cognitive styles or “brain types”, as measured by questionnaires. It was found that students in the Physical Sciences scored higher on the SQ-R and lower on the EQ in comparison to students the Humanities [12]. They on average showed Type S (S > E) cognitive style, while the Humanities students on average showed Type E (E > S) cognitive style [19–20].

As well as a theory of individual differences, the E-S theory is a theory of sex differences. It assumes that males and females have different brain structures that drive more females towards empathy and more males toward systemizing. Substantial amounts of data suggest that the brains of females and males are structurally different, A recent meta-analysis of neuroscience research of the last 20 years shows that males on average have larger total brain volumes than females, a difference that is evident from infancy throughout life. Differences in volume that were increased between females and males are located in specific brain areas [21]. In addition, studies that measured white matter in the brain show that the brains of women are wired differently than the brains of men. While men’s brains are on average significantly more interconnected across areas of the same hemisphere, brains of women are on average more interconnected between hemispheres [22].

These neurological differences may account, at least in part, for the behavioral and functional differences that are found on average between the sexes. Males on average scored higher on the SQ and females on average scored higher on the EQ [3, 4]. Males were more likely to have a Type S (S > E) cognitive style and females Type E (E > S) cognitive style [12, 15, 19, 23–27]. Moreover, EQ was correlated with emotional skills, such as the Reading the Mind in the Eyes’ test [19] and the Social Skills Inventory [28]. The SQ-R was correlated with analytic skills, such as embedded figures test [28] and mental rotation test [29, 30]. These correlations support the construct validity of the drives to systemize and empathize. Furthermore, independently of the E-S theory, but in line with it, differences between females and males were found in various tasks that considered to measure analytic versus emotional skills: males scored higher on mental rotation tasks [31, 32], navigation tasks [33–37], mathematical tests [38, 39] and females scored higher on verbal fluency [40], facial recognition [41], and other emotional tests [42–44].

The aim of the present study was to replicate the Billington et al. [19] study in light of the E-S theory. We assumed that the choice of major reflects a psychological tendency and we wanted to test if students in the Physical Sciences differ in their empathizing relative to systemizing from students in the Humanities. More specifically, we hypothesized that students in the Physical Sciences would score higher on the SQ-R relative to the EQ, and would thus more likely to be sorted into Type S brain (show a (S > E) profile). We also hypothesized that students in the Humanities would score higher on the EQ than on the SQ-R, and would thus be more likely to be sorted into a Type E brain (show a (E > S) profile). We predicted that male and female students on average would differ in their D scores, and in particular that more males would be sorted into Type S brain (show the S > E profile) and more females would be sorted into Type E brain (show the E > S profile). The question of whether the difference between the scores on the EQ and SQ-R is a better predictor of choice of academic degree than sex remains an open one.

## Method and materials

### Ethics statement

This study was approved by the "Touro College Institutional Review Board #1" and the “Stevens Institutional Review Board”. Participation was anonymous and voluntarily. Written informed consent was obtained from each participant.

### Participants

419 undergraduate students from two colleges located in the New York metropolitan area participated in this study. One college is a college of technology, where most of the students major in science, technology and engineering but few major in Humanities. The second is a college for Arts and Sciences, which offers degrees in liberal arts, social sciences, physical sciences, (including mathematics, computer science, and biology), but not in engineering. Out of the total of 419 students involved in the study, 147 were enrolled in the Humanities–116 of them were females and 31 were males. 272 were Science major students–120 of them were females and 152 males. All participants were between 20–25 years of age. The average age was 20 years and 6 months (*SD* = 1.7).

Recruitment was carried out via email and advertisement throughout the colleges. Research assistants handed out hard copy questionnaires throughout the classrooms to students of all majors. Each student received a set of all 3 questionnaires but the order was counterbalanced.(explain here) The entire process of announcement and distribution was designed to comply with IRB request for voluntary participation. Students could not fill out the questionnaires in the classrooms, they were asked to fill them out on their own time, and to drop them off in a drop-off box. Approximately 800 questionnaires were distributed within two weeks period. The entire process of distribution and collection took three weeks in one college and over a month in the other. 472 students returned questionnaires. Responses were recorded for statistical analysis by research assistants. 53 students did not respond to one or more questions so their data was excluded from analysis.

Our study was intended to test whether the choice of major would be correlated with the tendency to systemizing or to empathizing. We assumed that the preference one has to study physical sciences or the humanities reflects one’s tendency to systemizing or to empathizing. In our society, our interest in these fields is institutionalized into achieving an academic degree if we want to develop a career. One is forced then to choose an institution for the pursuit of one’s interest. Still, according to the E-S theory, the choice of major is specific to a person’s cognitive style, but not the choice of institution. Hence, the choice of major is an important variable in our study, while the choice of college is not. Indeed one can have a preference to the humanities or the sciences without being conferred with a degree; the E-S theory prediction would still be the same. Therefore, the colleges our participants were attending, each offered majors in the sciences and humanities, were only a channel through which to collect data and not a variable of comparison. Therefore, we treated whatever differences between the colleges as irrelevant to the purpose of this study and its theoretical framework.

Academic majors were divided into two categories: Humanities and Physical Sciences. Physical Sciences included the mathematics, physics, engineering, computer science, biology, actuarial science, finance, chemistry, and accounting. Humanities included the psychology, education, art, music, business, speech therapy, and political science. Similarly, biological sex was relevant for our aim and therefore was collected any other information regarding the subject was ignored (e.g. ethnicity, religion or race). Biological sex was divided into males and females.

### Measures

The Empathy Questionnaire (EQ) is a self-report, 40-statement questionnaire that measures levels of two aspects of empathy, cognitive and affective. The cognitive aspect of empathy is the ability to understand mental states of others. The affective aspect is the drive to respond to a person’s mental state with an appropriate emotion. 21 statements depict empathic situations (e.g., *“I am good at predicting how someone will feel”*) and 19 describe the opposite (e.g., *“I can't always see why someone should have felt offended by a remark”*). All 40 statements are rated on 4-point Likert scale ranging from ‘strongly agree’ to ‘strongly disagree’. Responses that align with empathy are scored 2 or 1, and responses that are non-empathic are scored 0. More specifically, on the 21 empathic statements a “definitely agree” gets 2 points, a “slightly agree” gets 1 point, and “slightly disagree” and “definitely disagree” each gets 0 points. On the 19 statements that depict non empathic situations, scoring is reversed and “strongly disagree” gets 2 points, “slightly disagree” gets 1 point and “slightly agree” and “strongly agree” both gets 0 points. The maximum possible total score is 80, and the lowest is 0. The Empathy Quotient (EQ) can be found in the supporting information (S1 Questionnaire) and at the Autistic Research Centre website https://www.autismresearchcentre.com/

The Systemizing Quotient-Revised (SQ-R) is a self-report, 75-statement questionnaire measuring systemizing skills (attention to details, recognizing patterns) in various systems. Responses are rated on a 4-point Likert scale, definitely disagree, slightly disagree, slightly agree, and definitely agree. On statements that are in agreement with systemizing skills (e.g., “*When I look at a building*, *I am curious about the precise way it was constructed”*), “definitely agree” gets 2 points, “slightly agree” gets 1 point, “slightly disagree” and “definitely disagree” each gets 0 points. On statements that describe the opposite tendency (e.g., “*I find it difficult to read and understand maps”*), scoring is reversed. The maximum possible total score is 150 and the lowest is 0. The Systemizing Quotient-Revised (SQ-R) can be found in the supporting information (S2 Questionnaire) and at the Autism Research Centre website https://www.autismresearchcentre.com/

The Academic Major Questionnaire includes questions on the student’s sex, date of birth, type of major and academic year.

### E-S discrepancy

To calculate how performances on the two scales stand relative to each other in the overall population, the difference (D) between the standardized SQ-R score (S) and the standardized EQ score (E) was used [15]. The standardized S and the standardized E are computed by the formulae S = (SQ-R—<SQ-R>) / 150 and E = (EQ—<EQ>) / 80 respectively. This means that S and E is each the result of subtraction the sample mean (denoted by < …>) from the individual raw score divided by the maximum score for the test, i.e., 150 for the SQ-R and 80 for the EQ. The difference (D) between the standardized EQ and SQ-R scores is then calculated by: D = (S—E) / 2. Positive D scores indicate a stronger tendency to systemizing relative to empathizing and vice versa. D scores close to zero represent an equal drive to systemize and empathize [15].

### Cognitive profiles or “brain types”

To calculate cognitive profiles or “brain types” the D scores are divided into 5 percentiles following previous procedure [15]. Individuals who score in the lowest 2.5^th^ percentile are defined as Extreme Type E (E >> S), those who scores between the 2.5^th^ and 35^th^ percentiles are classed as Type E (E > S), those between the 35^th^ and 65^th^ percentile as Type B (balanced, E ≈ S), between the 65^th^ and 97.5^th^ percentile as Type S (S > E), and those who scored in highest 2.5^th^ percentiles are Extreme Type S (S >> E).

## Results

Table 1 shows the means and standard deviations of EQ and R-SQ and D scores for males and females in the humanities and the sciences.

**Table 1:** Means and standard deviations of EQ and SQ-R and D scores for males and females in the humanities and the sciences.

Sex	Major		EQ Scale		SQ-R Scale		D Scores	
		*N*	*M*	*SD*	*M*	*SD*	*M*	*SD*
**Females**	Humanities	116	51.0	11.8	50.1	17.2	-0.084	0.090
	Sciences	120	44.1	11.6	66.9	19.2	0.015	0.093
**Males**	Humanities	31	43.5	12.6	64.6	18.8	0.012	0.080
	Sciences	152	39.0	11.8	67.5	18.8	0.050	0.082

*M*: mean; *SD*: standard deviation.

As can be seen from Table 1, the number of males in the Humanities is relatively small, which may affect the 2-way ANOVAs below. Therefore we ran statistical procedures to check if ANOVA assumption were violated. The Shapiro-Wilk normality test was not significant (*W* = 0.99, *p* = 0.73), meaning normality was not violated. A Levene's test for homogeneity of variance (center = median) conducted on D scores was not significant (F(105, 313) = 0.97, *p*>0.58). In addition a Levene's test for homogeneity of variance (center = median) conducted on the EQ and SQ-R raw scores and on the interaction Sex by Major was not significant (*p* > 0.1), meaning homogeneity of variance was not violated.

**Fig 1:**
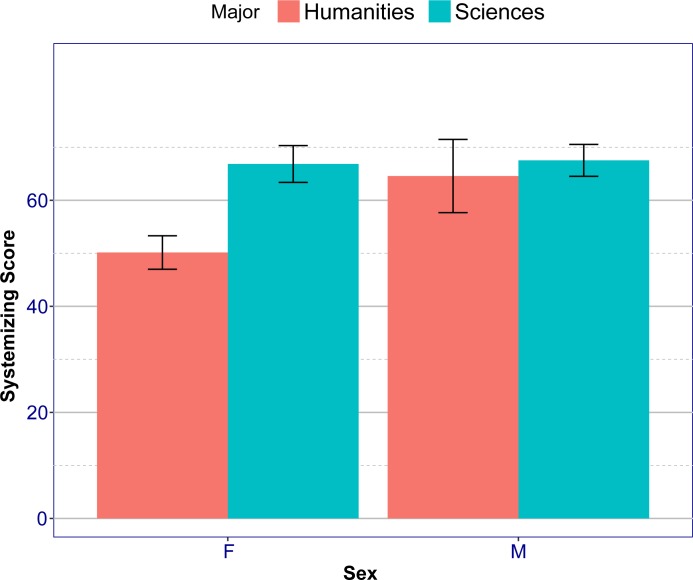
Systemizing scores as a function of sex and major. Error bars show 95% confidence intervals.

A 2X2 ANOVA on the raw systemizing scores with Sex and Major as factors revealed significant main effects to Sex (*F*(1, 415) = 21.3, *p =* 5.3·10^−6^, *η*_*p*_^2^ = .01), Major (*F*(1, 415) = 39.0, *p =* 1.1·10^−9^, *η*_*p*_^2^ = .09) and an interaction (*F*(1, 415) = 9.8, *p* = 0.0018, *η*_*p*_^2^ = 0.02). A post-hoc Tukey analysis on the raw systemizing scores revealed that females in the Humanities scored significantly lower on the SQ-R relative to each one of the other three groups (males in the Sciences (*p* < 2·10^−16^), females in the Sciences (*p* < 2·10^−16^) and males in the Humanities (*p* = 7.5·10^−4^)). The scores on the SQ-R between the other three groups were not significantly different (*p* > .8 for all of them) (Fig 1).

**Fig 2:**
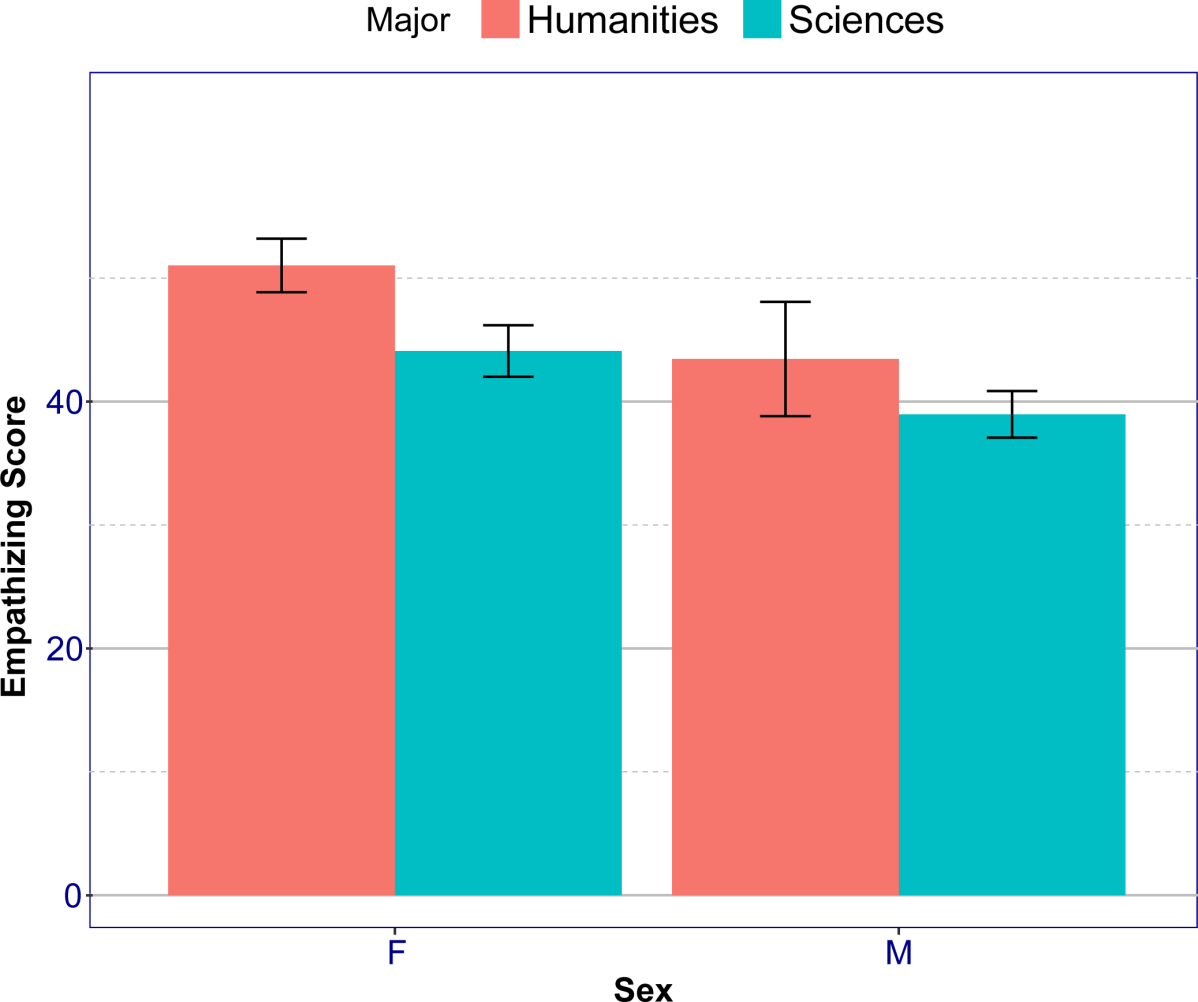
Empathizing scores as a function of sex and major. Error bars show 95% confidence intervals.

A 2X2 ANOVA on raw empathizing scores with Sex and Major as factors revealed a significant main effect of Sex (*F* (1, 415) = 44.9, *p* = 6.7·10^−11^, *η*_*p*_^2^ = .05) and Major (*F*(1, 415) = 23.4, *p* = 1.9·10^−6^, *η*_*p*_^2^ = .05), but no interaction (*F*(1, 415) = 0.8, *p* = 0.38, *η*_*p*_^2^ = 0.002) (Fig 2). A post-hoc Tukey analysis on the raw empathizing scores revealed that females in the Humanities scored significantly higher on the EQ than males in the Humanities (*p* = .0086). They scored significantly higher than females in the Sciences (*p* = 4.9·10^−5^) and males in the Sciences (*p* < 2·10^−16^). Females in the Sciences scored significantly higher than males in the Sciences (*p* = .0023). No other significant differences were found (*p* >.2) (Fig 2).

**Fig 3:**
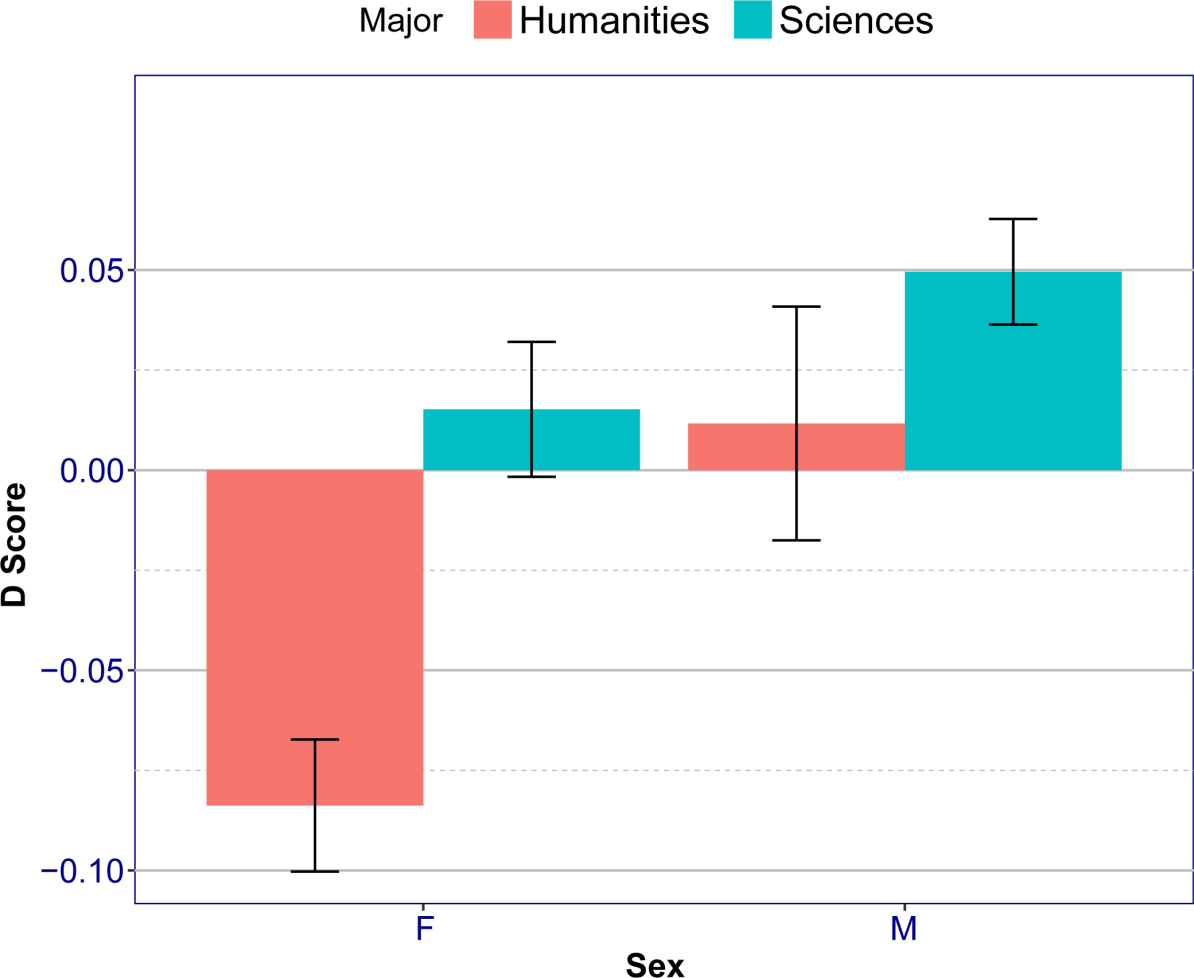
D scores as a function of sex and major. Error bars show 95% confidence intervals.

A 2X2 ANOVA using D scores (E-S discrepancy) as the dependent measure and the type of Major (Sciences vs. Humanities) and Sex (female vs. male) as two independent variables was conducted on the data. ANOVA analysis revealed significant main effects of Sex (*F*(1, 415) = 79.1, *p* < 2·10^−16^, *η*_*p*_^2^ = .07), Major (*F*(1, 415) = 71.7, *p* = 4.4·10^−16^, *η*_*p*_^2^ = .14) and a significant interaction (*F*(1, 415) = 8.8, *p* = .0033, *η*_*p*_^2^ = 0.02). Results are shown in Fig 3. As can be seen, D score is on average higher for males than females irrespective of their major, and is on average higher for Sciences than Humanities, irrespective of their sex. D score is on average lowest for females in the Humanities. A post-hoc Tukey analysis on D scores revealed a significant difference between males (MH) and females in the Humanities (FH) (*p* = 7.0·10^−7^), between females in Sciences (FS) and females in the Humanities (FH) (*p* < 2·10^−16^), between males in Sciences (MS) and females in Humanities (FH) (*p* < 2·10^−16^), between males in Sciences (MS) and females in Sciences (FS) (*p* = .0076). There was no significant difference between females in Sciences (FS) and males in Humanities (MH), or between males in Sciences (MS) and males in Humanities (MH) (*p* > 0.10).

**Fig 4:**
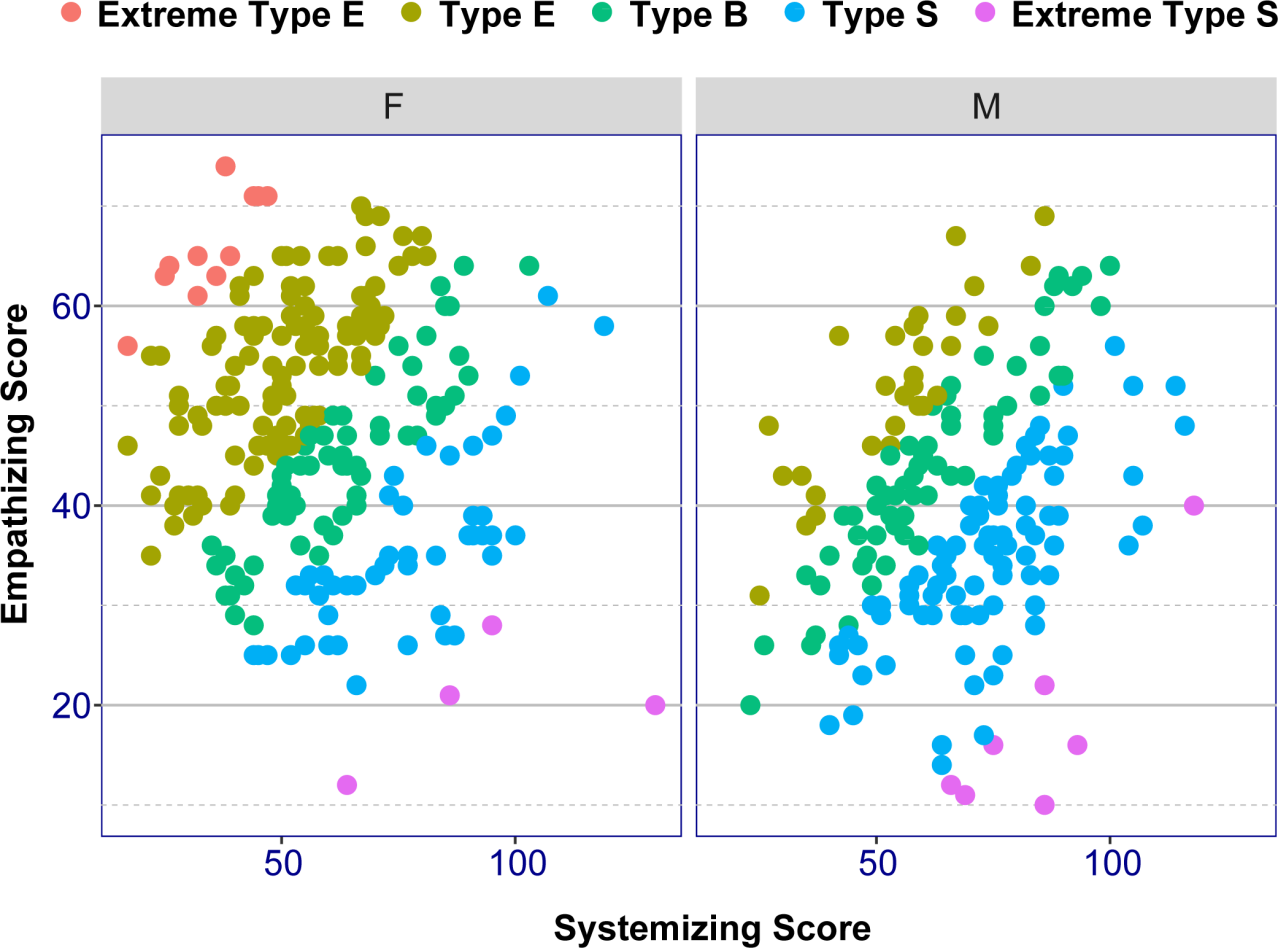
Distributions of D scores across “brain types” for females (F) and for males (M).

D scores were separated into 5 quintiles. Each quintile represents one of the 5 “brain types”. Table 2 shows the boundary for each “brain type” calculated from D scores for current sample. Fig 4 shows the distributions of D scores across “brain types”. D scores run from the lowest (top left corner), to the highest (bottom right corner). Table 3 shows the number of participants classed in each “brain type” by Sex, Major and combined Sex-Major. It also shows all percentages by “brain type” and by groups which help explain the study topics. For example, given Extreme Type S, we can see the distribution for Sex or Major, or both.

**Table 2:** Brain type boundaries, based on D scores of the current sample.

Brain Types	Brain type boundaries
Extreme E	D < -0.20
Type E	-0.20 ≤ **D** < -0.04
Type B	-0.041 ≤ **D** < 0.043
Type S	0.043 ≤ **D** < 0.21
Extreme S	D ≥ 0.21

**Table 3:** Percentage of participants classed into each brain type by sex, major and both sex and major.

Brain Type	Sex		Major		Sex and Major			
	Females	Males	Humanities	Sciences	FH	MH	FS	MS
	**Numbers**							
**Extreme E**	11	0	9	0	11	0	0	0
**Type-E**	107	29	79	57	71	8	36	21
**Type-B**	65	60	35	90	23	12	42	48
**Type-S**	49	87	21	115	10	11	39	76
**Extreme S**	4	7	1	10	1	0	3	7
	**Percentages within each brain type by group**							
**Extreme E**	100%	0%	100%	0%	100%	0%	0%	0%
**Type-E**	79%	21%	58%	42%	52%	6%	26%	16%
**Type-B**	52%	48%	28%	72%	18%	10%	34%	38%
**Type-S**	36%	64%	15%	85%	7%	8%	29%	56%
**Extreme S**	36%	64%	9%	91%	9%	0%	27%	64%
	**Percentages within each group by brain type**							
**Extreme E**	5%	0%	7%	0%	9%	0%	0%	0%
**Type-E**	45%	16%	54%	21%	61%	26%	30%	14%
**Type-B**	28%	33%	24%	33%	20%	39%	35%	32%
**Type-S**	21%	48%	14%	42%	9%	35%	32%	50%
**Extreme S**	2%	4%	1%	4%	1%	0%	3%	5%

FH = females in Humanities, MH = males in Humanities, FS = females in Sciences, MS = males in the Sciences.

As can be seen from Table 3, more females than males were classed as Type E and Extreme Type E. More males than females were classified as Type S and Extreme Type S. More Humanities majors than Sciences majors were classed as Type E and Extreme Type E, and more Sciences majors than Humanities majors were classified as Type S and Extreme Type S. More females in the Humanities were classified as Type E and Extreme Type E relative to males in the Humanities, females in the Sciences, and males in the sciences. More males in the Sciences were classified as Type S and Extreme Type S compared to females in the Sciences and male and females in the Humanities. This pattern of distribution was confirmed by Fisher exact tests of independence, which revealed that the relationship between sex and “brain type” was highly significant (*p* = 7.4·10^−14^), the relationship between major and “brain type” was highly significant (*p* < 2·10^−16^) and the relationship between combined sex-major and “brain type” was highly significant (*p* < 2·10^−16^). Fisher exact test was used as a post-hoc analysis. Comparisons were done on the columns between “brain types” and on the rows between females versus males, Humanities versus Sciences and between the four groups of combined sex-major. Bonferroni method was used to adjust *p* values. Results are shown in Table 4.

**Table 4:** Fisher exact test as a post-hoc analysis.

**Fisher Exact comparisons for Sex**	
Comparison Between Brain Types (Columns)	Adjusted ***p* value**
Extreme E vs. Type-E	1.0000
Extreme E vs. Type-B	0.0125
Extreme E vs. Type-S	0.0003
Extreme E vs. Extreme S	0.0387
Type-E vs. Type-B	0.0001
Type-E vs. Type-S	0.0000
Type-E vs. Extreme S	0.0485
Type-B vs. Type-S	0.1235
Type-B vs. Extreme S	1.0000
Type-S vs. Extreme S	1.0000
Females vs. Males (row)	0.0000
**Fisher Exact comparisons for Majors**	
Comparison Between Brain Types (Columns)	Adjusted ***p* value**
Extreme E vs. Type-E	0.0691
Extreme E vs. Type-B	0.0000
Extreme E vs. Type-S	0.0000
Extreme E vs. Extreme S	0.0003
Type-E vs. Type-B	0.0000
Type-E vs. Type-S	0.0000
Type-E vs. Extreme S	0.0267
Type-B vs. Type-S	0.1580
Type-B vs. Extreme S	1.0000
Type-S vs. Extreme S	1.0000
Humanities vs. Sciences (row)	0.0000
**Fisher Exact comparisons for both Sex and Major**	
Comparison Between Brain Types (Columns)	Adjusted ***p* value**
Extreme E vs. Type-E	0.3073
Extreme E vs. Type-B	0.0000
Extreme E vs. Type-S	0.0000
Extreme E vs. Extreme S	0.0003
Type-E vs. Type-B	0.0000
Type-E vs. Type-S	0.0000
Type-E vs. Extreme S	0.0172
Type-B vs. Type-S	0.1042
Type-B vs. Extreme S	1.0000
Type-S vs. Extreme S	1.0000
Females Humanities vs. Males Humanities (row)	0.0002
Females Humanities vs. Females Science (row)	0.0000
Females Humanities vs. Males Science (row)	0.0000
Males Humanities vs. Female Science (row)	1.0000
Males Humanities vs. Males Science (row)	0.9931
Females Science vs. Males Science (row)	0.0144

### Discussion

This study tested the relationship between sex, major and D scores (classified as one of 5”brain types” defined by the magnitude of the discrepancy between EQ scores and SQ-R scores). Our study shows that on average males score higher on systemizing relative to empathizing, and on average females score higher on empathizing relative to systemizing. It also shows that on average Sciences students have higher systemizing than empathizing scores, and Humanities students on average have higher empathizing than systemizing scores. The E-S theory of sex differences proposes that females on average are more driven to empathize and males on average are more driven to systemize, and therefore hypothesizes that in a random population females will on average score higher on the EQ and males on average will score higher on the SQ-R. Our results are consistent with these predictions and replicate Billington et al. [19] results and those of others [3, 4, 12, 15, 20, 23, 25–27]. In addition, finding a difference between scores on the EQ and scores on SQ-R within academic degree replicates the Billington et al. [19] and is in line with other studies [12, 20, 24]. The EQ scale and SQ-R scale in our data have similar means and standard deviations to that of Billington et al. [19]. Sample size, group size (including the relatively smaller size of males in Humanities) are also very similar (Billington et al. [19] study sample size, groups size, means and standard deviations are shown in S1 Table in the supporting information).

The present study shows clear sex and major effects on the 5 “brain types”, which supports the E-S theory hypothesis that in a typical population, more females on average are sorted into Type E and Extreme Type E and more males on average are sorted into Type S and Extreme Type S. The current pattern of distribution of D scores across the 5 “brain types” is very similar to what has been found in previous studies [12, 15, 23]. In relation to academic degree, the present study shows that only female students of the Humanities were classed in the Extreme Type E brain, and the majority of this group was classed into Type E brain. In contrast, the majority of Sciences male students were classed into Type S and Extreme Type S.

Unlike Billington et al. [19] study and studies of others [12, 20, 24], we found a significant interaction between sex and major on D scores. Scoring higher or lower on E-S discrepancy (D scores) in the Humanities versus the Sciences is sex-dependent. Females in the Humanities show a significantly stronger tendency to empathize than to systemize compared to their male or female counterparts in the Humanities or in the Sciences. Males in the Sciences show a significantly stronger tendency to systemize than to empathize compared to their female counterparts in the Sciences or Humanities. However, the difference between females and males in the Sciences is smaller in comparison to the difference between females and males in the Humanities. This suggests that the driving force of the interaction may come from the latter.

What may account for the presence of the interaction in our study and the lack of it in Billington et al. [19] study are the different types of majors that were included in each of the studies. In the Billington et al. [19] study the majors in the Humanities were: philosophy, English, history, music, education, drama, languages, linguistics, classics, architecture, law, theology, oriental studies, Anglo-Saxon and Celtic studies, history, philosophy of science and the history of art. In our study the majors were: psychology, education, art, music, business, speech therapy, and political sciences. In the Billington et al. [19] study the majors in the Sciences were: mathematics, physics, physical natural sciences, chemistry, computer science, geology, communications, engineering, manufacturing engineering, chemical engineering, mineral science, material science, astrophysics, astronomy, and geophysics. In our study the majors were: mathematics, physics, engineering, computer science, biology, actuarial science, finance, chemistry and accounting. Billington et al. [19] included a wider range of majors and different ones. It is possible that the aggregation of scores across the majors cancels out the E-S discrepancy between females and males in Billington et al. [19] study. In contrast, the composition of majors in our study brings out the interaction that we find here. This implies that the D scores are distributed differently across majors. Further research should compare between majors specifically.

The current finding suggest that females who are driven more by empathizing and who are classified into Type E brain or Extreme Type E brain based on their D scores are more likely to be attracted to the Humanities as a major of preference. The Sciences, however, are likely to be the degree of choice for individuals that are classified into Type S brain or Extreme Type S brain, and sex plays a lesser role in this choice. Being sorted to one “brain type” and not another, might explain, at least in part, the sex differences found in academia, whereby although women outnumber men in undergraduate enrollment in a ratio of 56–44 [45], females are more attracted to Humanities than to Sciences. In 2010 more Bachelor’s degrees in psychology, education, and health profession were awarded to women than to men, and more Bachelor’s degrees in engineering, computer science, and physical sciences were awarded to men than to women [46, 47]. In 2014, more women were awarded a Bachelor’s degree in psychology, anthropology and sociology than men, and more men were awarded a Bachelor’s degree in engineering and computer science than women [47].

According to the E-S theory, D scores show a range of discrepancies between empathizing and systemizing across a general population. D scores with smaller discrepancies concentrate in the middle and gradually negative and positive D scores disperse on opposite sides. D scores of females or males can fall anywhere along the D continuum, but the proportion of females decreases as D scores become more positive and proportion of males decreases as D scores become more negative. On average more males will show more positive D scores and more females will show more negative D scores. In addition, more individuals with increasingly positive D scores are concentrated in subjects that require more systemizing. Our data shows that more positive D score, or Type S brain, is associated more with the Sciences and that this association is less affected by sex. It suggests that females or males higher on systemizing and lower on empathizing are more likely to be attracted to the Sciences. However, our data also shows that males achieve higher D score than females and that more males are sorted into Type S brain. This finding is in agreement with the finding that women earn Bachelor’s degree in engineering, mathematics, statistics, physics and biology but the overall proportion is small [48]. The proportion of women also varies between fields; for example women are represented in biology more than in engineering [47]. How the differences between females and males on the D scores are related to specific choice of major within the Sciences needs to be determined by further research.

Our study shows that a group of males were sorted into Type S brain in the Humanities, which may seem counter-intuitive. However, the E-S theory claims that there is a range of systems, including social and abstract ones that might appeal to individuals higher on systemizing. Certain majors in the Humanities involve such systems. Our study cannot point out in what majors the higher systemizing males were enrolled as we treated the Humanities as one group. However, an indication that males sorted into Type S brain are more likely to be associated with system-based majors in the Humanities is that the proportion of males and females varies across majors in the Humanities. For example, more males study philosophy than females [49], more females study psychology than males [47, 50]. Philosophy may require relatively more systemizing than empathizing and psychology may involve relatively more empathizing than systemizing. Future research should study if such sex-related differences in the choice of major in the Humanities are related to the way D scores are distributed among females and males across majors in the Humanities.

It is possible that other psychological and social factors play a role in the choice of majors beside the drive to systemizing. For example, one’s self efficacy to perform in a certain field may influence one’s decision. Level of identification with the subject might also play a role. In addition, efforts to promote the Sciences to females might be fruitful in attracting females higher on systemizing to the Sciences.

Whilst we recognize that social factors (such as the absence of senior female role models in the exact sciences, or teacher-, parent-, media-, and peer-led socialization processes) also contribute to such choices, the current study also suggests that such degree choices are also influenced by one’s cognitive brain type, which itself is shaped by both social factors and prenatal biology [2]. The E-S theory proposes that the differences between females and males in the drive to systemize and empathize stems in part from prenatal biological factors, whilst acknowledging that prenatal biology interacts with postnatal social experience. The present study was not designed to study this question but the genetics and prenatal endocrinology of empathy and systemizing are under investigation elsewhere [8–11, 51, 52].

One limitation of this study is that it only used self-report questionnaires. A person’s behavior and how that person reports on their own behavior can be already influenced by that person’s background, socialization, as well as having already made a choice, and the culture of the academic fields one’s is already in. Future studies therefore need to confirm E-S theory using performance tests, rather than solely relying on self-report measures. Another limitation is smaller group size of the Humanities, which was due to lower response rate. And also the smaller group size of males in the Humanities, which was due to a combination of low enrollment of men in the Humanities, and fewer males in the Humanities completing the questionnaires. However, there was no violation of normality and homogeneity of variance. Future studies could test these patterns of distribution using much larger samples collected online. Despite these limitations, the current study is exactly in line with results from previously reported studies, across a range of cultures, suggesting predictions from the E-S theory reflect universal dimensions of the human mind, independent of culture.

## Supporting information

S1 Questionnaire The Empathy Questionnaire (EQ). (DOC)

S2 Questionnaire The Systemizing Quotient-Revised (SQ-R). (DOC)

S1 Table Billington et al. [19] study, means and standard deviations of EQ and SQ-R for males and females in the humanities and the sciences. (DOC)

S1 Dataset Data summary. (XLS)
