# Supplementary material for: Empathizing-systemizing cognitive styles: Effects of sex and academic degree
Source: PLoS One. 2018 Mar 26;13(3):e0194515. doi: 10.1371/journal.pone.0194515 (PMC5868797; doi:10.1371/journal.pone.0194515)
Supplement: S2 Questionnaire — (DOC) [file pone.0194515.s002.doc]

**Revised Cambridge Personality Questionnaire**

*Please fill in this information and then read the instructions below.*

**ALL INFORMATION REMAINS STRICTLY CONFIDENTIAL**

Name:........................................................................ Sex:.........................

Date of birth:.................................... Today’s date:..................................

**How to fill out the questionnaire**

Below is a list of statements. Please read each statement very carefully and rate how strongly you agree or disagree with it by typing an ‘X’ in the appropriate box. There are no right or wrong answers, or trick questions.

**IN ORDER FOR THE SCALE TO BE VALID, YOU MUST ANSWER EVERY QUESTION.**

|  |  | strongly  agree | slightly  agree | slightly  disagree | strongly  disagree |
| --- | --- | --- | --- | --- | --- |
| 1. | I find it very easy to use train timetables, even if this involves several connections. |  |  |  |  |
| 2. | I like music or book shops because they are clearly organised. |  |  |  |  |
| 3. | I would not enjoy organising events e.g. fundraising evenings, fetes, conferences. |  |  |  |  |
| 4. | When I read something, I always notice whether it is grammatically correct. |  |  |  |  |
| 5. | I find myself categorising people into types (in my own mind). |  |  |  |  |
| 6. | I find it difficult to read and understand maps. |  |  |  |  |
| 7. | When I look at a mountain, I think about how precisely it was formed. |  |  |  |  |
| 8. | I am not interested in the details of exchange rates, interest rates, stocks and shares. |  |  |  |  |
| 9. | If I were buying a car, I would want to obtain specific information about its engine capacity. |  |  |  |  |
| 10. | I find it difficult to learn how to programme video recorders. |  |  |  |  |
| 11. | When I like something I like to collect a lot of different examples of that type of object, so I can see how they differ from each other. |  |  |  |  |
| 12. | When I learn a language, I become intrigued by its grammatical rules. |  |  |  |  |
| 13. | I like to know how committees are structured in terms of who the different committee members represent or what their functions are. |  |  |  |  |
| 14. | If I had a collection (e.g. CDs, coins, stamps), it would be highly organised. |  |  |  |  |
| 15. | I find it difficult to understand instruction manuals for putting appliances together. |  |  |  |  |
| 16. | When I look at a building, I am curious about the precise way it was constructed. |  |  |  |  |

|  |  | strongly  agree | slightly  agree | slightly  disagree | strongly  disagree |
| --- | --- | --- | --- | --- | --- |
| 17. | I am not interested in understanding how wireless communication works (e.g. mobile phones). |  |  |  |  |
| 18. | When travelling by train, I often wonder exactly how the rail networks are coordinated. |  |  |  |  |
| 19. | I enjoy looking through catalogues of products to see the details of each product and how it compares to others. |  |  |  |  |
| 20. | Whenever I run out of something at home, I always add it to a shopping list. |  |  |  |  |
| 21. | I know, with reasonable accuracy, how much money has come in and gone out of my bank account this month. |  |  |  |  |
| 22. | When I was young I did not enjoy collecting sets of things e.g. stickers, football cards etc. |  |  |  |  |
| 23. | I am interested in my family tree and in understanding how everyone is related to each other in the family. |  |  |  |  |
| 24. | When I learn about historical events, I do not focus on exact dates. |  |  |  |  |
| 25. | I find it easy to grasp exactly how odds work in betting. |  |  |  |  |
| 26. | I do not enjoy games that involve a high degree of strategy (e.g. chess, Risk, Games Workshop). |  |  |  |  |
| 27. | When I learn about a new category I like to go into detail to understand the small differences between different members of that category. |  |  |  |  |
| 28. | I do not find it distressing if people who live with me upset my routines. |  |  |  |  |
| 29. | When I look at an animal, I like to know the precise species it belongs to. |  |  |  |  |
| 30. | I can remember large amounts of information about a topic that interests me e.g. flags of the world, airline logos. |  |  |  |  |
| 31. | At home, I do not carefully file all important documents e.g. guarantees, insurance policies |  |  |  |  |
| 32. | I am fascinated by how machines work. |  |  |  |  |
| 33. | When I look at a piece of furniture, I do not notice the details of how it was constructed. |  |  |  |  |

|  |  | strongly  agree | slightly  agree | slightly  disagree | strongly  disagree |
| --- | --- | --- | --- | --- | --- |
| 34. | I know very little about the different stages of the legislation process in my country. |  |  |  |  |
| 35. | I do not tend to watch science documentaries on television or read articles about science and nature. |  |  |  |  |
| 36. | If someone stops to ask me the way, I'd be able to give directions to any part of my home town. |  |  |  |  |
| 37. | When I look at a painting, I do not usually think about the technique involved in making it. |  |  |  |  |
| 38. | I prefer social interactions that are structured around a clear activity, e.g. a hobby. |  |  |  |  |
| 39. | I do not always check off receipts etc. against my bank statement. |  |  |  |  |
| 40. | I am not interested in how the government is organised into different ministries and departments. |  |  |  |  |
| 41. | I am interested in knowing the path a river takes from its source to the sea. |  |  |  |  |
| 42. | I have a large collection e.g. of books, CDs, videos etc. |  |  |  |  |
| 43. | If there was a problem with the electrical wiring in my home, I’d be able to fix it myself. |  |  |  |  |
| 44. | My clothes are not carefully organised into different types in my wardrobe. |  |  |  |  |
| 45. | I rarely read articles or webpages about new technology. |  |  |  |  |
| 46. | I can easily visualise how the motorways in my region link up. |  |  |  |  |
| 47. | When an election is being held, I am not interested in the results for each constituency. |  |  |  |  |
| 48. | I do not particularly enjoy learning about facts and figures in history. |  |  |  |  |
| 49. | I do not tend to remember people's birthdays (in terms of which day and month this falls). |  |  |  |  |
| 50. | When I am walking in the country, I am curious about how the various kinds of trees differ. |  |  |  |  |
| 51. | I find it difficult to understand information the bank sends me on different investment and saving systems. |  |  |  |  |
| 52. | If I were buying a camera, I would not look carefully into the quality of the lens. |  |  |  |  |

|  |  | strongly  agree | slightly  agree | slightly  disagree | strongly  disagree |
| --- | --- | --- | --- | --- | --- |
| 53. | If I were buying a computer, I would want to know exact details about its hard drive capacity and processor speed. |  |  |  |  |
| 54. | I do not read legal documents very carefully. |  |  |  |  |
| 55. | When I get to the checkout at a supermarket I pack different categories of goods into separate bags. |  |  |  |  |
| 56. | I do not follow any particular system when I'm cleaning at home. |  |  |  |  |
| 57. | I do not enjoy in-depth political discussions. |  |  |  |  |
| 58. | I am not very meticulous when I carry out D.I.Y or home improvements. |  |  |  |  |
| 59. | I would not enjoy planning a business from scratch to completion. |  |  |  |  |
| 60. | If I were buying a stereo, I would want to know about its precise technical features. |  |  |  |  |
| 61. | I tend to keep things that other people might throw away, in case they might be useful for something in the future. |  |  |  |  |
| 62. | I avoid situations which I can not control. |  |  |  |  |
| 63. | I do not care to know the names of the plants I see. |  |  |  |  |
| 64. | When I hear the weather forecast, I am not very interested in the meteorological patterns. |  |  |  |  |
| 65. | It does not bother me if things in the house are not in their proper place. |  |  |  |  |
| 66. | In maths, I am intrigued by the rules and patterns governing numbers. |  |  |  |  |
| 67. | I find it difficult to learn my way around a new city. |  |  |  |  |
| 68. | I could list my favourite 10 books, recalling titles and authors' names from memory. |  |  |  |  |
| 69. | When I read the newspaper, I am drawn to tables of information, such as football league scores or stock market indices. |  |  |  |  |
| 70. | When I’m in a plane, I do not think about the aerodynamics. |  |  |  |  |
| 71. | I do not keep careful records of my household bills. |  |  |  |  |
| 72. | When I have a lot of shopping to do, I like to plan which shops I am going to visit and in what order. |  |  |  |  |

|  |  | strongly  agree | slightly  agree | slightly  disagree | strongly  disagree |
| --- | --- | --- | --- | --- | --- |
| 73. | When I cook, I do not think about exactly how different methods and ingredients contribute to the final product. |  |  |  |  |
| 74. | When I listen to a piece of music, I always notice the way it’s structured. |  |  |  |  |
| 75. | I could generate a list of my favourite 10 songs from memory, including the title and the artist's name who performed each song. |  |  |  |  |

Thank you for completing this questionnaire.

©SBC/SJW Nov 2003
