# Supplementary material for: Empathizing-systemizing cognitive styles: Effects of sex and academic degree
Source: PLoS One. 2018 Mar 26;13(3):e0194515. doi: 10.1371/journal.pone.0194515 (PMC5868797; doi:10.1371/journal.pone.0194515)
Supplement: S1 Table — (DOC) [file pone.0194515.s003.doc]

**S1Table. Billington et al. [19] Study, Means and Standard Deviations of EQ**

**and SQ-R for Males and Females in the Humanities and the Sciences**

| **Sex** | **Major** |  | **EQ Scale** | | **SQ-R Scale** | |
| --- | --- | --- | --- | --- | --- | --- |
|  |  | ***N*** | ***M*** | ***SD*** | ***M*** | ***SD*** |
| **Females** | Humanities | 104 | 46.82 | 12.07 | 51.54 | 19.18 |
|  | Sciences | 108 | 43.48 | 12.56 | 61.23 | 20.60 |
| **Males** | Humanities | 43 | 40.56 | 10.33 | 58.65 | 21.17 |
|  | Sciences | 160 | 35.59 | 10.39 | 65.46 | 18.17 |

*M*: mean; *SD*: standard deviation.
